# Melatonin protects hippocampal HT22 cells from the effects of serum deprivation specifically targeting mitochondria

**DOI:** 10.1371/journal.pone.0203001

**Published:** 2018-08-29

**Authors:** Erica Cesarini, Liana Cerioni, Barbara Canonico, Gianna Di Sario, Andrea Guidarelli, Davide Lattanzi, David Savelli, Michele Guescini, Maria Gemma Nasoni, Noemi Bigini, Riccardo Cuppini, Vilberto Stocchi, Patrizia Ambrogini, Stefano Papa, Francesca Luchetti

**Affiliations:** Department of Biomolecular Sciences, University of Urbino Carlo Bo, Urbino, Italy; National Research Council, ITALY

## Abstract

Neurons contain a high number of mitochondria, these neuronal cells produce elevated levels of oxidative stress and live for a long time without proliferation; therefore, mitochondrial homeostasis is crucial to their health. Investigations have recently focused on mitochondrial dynamics revealing the ability of these organelles to change their distribution and morphology. It is known that mitochondrial fission is necessary for the transmission of mitochondria to daughter cells during mitosis and mitochondrial fragmentation has been used as an indicator of cell death and mitochondrial dysfunction. Oxidative stress is a trigger able to induce changes in the mitochondrial network. The aim of the present study was to determine the effects of melatonin on the mitochondrial network in HT22 serum-deprived cells. Our results showed that serum deprivation increased reactive oxygen species (ROS) content, promoted the activation of plasma membrane voltage-dependent anion channels (VDACs) and affected the expression of pDRP1 and DRP1 fission proteins. Moreover, parallel increases in apoptotic and autophagic features were found. Damaged and dysfunctional mitochondria are deleterious to the cell; hence, the degradation of such mitochondria through mitophagy is crucial to cell survival. Our results suggest that melatonin supplementation reduces cell death and restores mitochondrial function through the regulation of autophagy.

## Introduction

Over the past few years, several authors have investigated the role of mitochondria in physiology and disease, mostly focusing on neurodegenerative diseases [1, 2]. Mitochondrial features and function are controlled by the morphological dynamics of these organelles, which migrate, divide and fuse. In particular, the maintenance of mitochondrial network is mediated by a correct balance between fusion and fission process [3]. Mitochondrial fission is orchestrated by dynamin-related protein1 (DRP1) [4], a GTPase protein of the dynamin family. A considerable body of evidence indicates that inhibition of DRP1 provides neuroprotection, whereas a loss of mitofusin2 (Mfn2) results in the degeneration of nigrostriatal dopaminergic neurons, showing that mitochondrial dynamics are closely associated with neuron death [5]. Despite the strong correlation between mitochondrial fission and cell death, some studies have questioned the importance of mitochondrial fission and fragmentation in apoptosis [6]. On the other hand, other investigations have suggested that mitochondrial fission contributes to chronic neurodegeneration through other non-apoptotic cell death pathways such as autophagic or necrosis-like pathways [7]. Mitochondria are the primary source of ROS generation as well as the major target of free radical attacking. Several authors have demonstrated the direct connections between oxidative stress and mitochondrial morphology in endothelial cells, in neurons and more recently in C2C12 cells [8, 9]. However, at present, it is uncertain whether mitochondrial fission induces oxidative stress or oxidative stress disrupts mitochondrial dynamics [10].

Fetal bovine serum (FBS) is essential for most *in vitro* cell cultures as it contains all the necessary element for cell growth [11]. Hence, when cells are grown in a serum deprived (SD) conditions they undergo apoptosis and autophagy. The latter generally functions as a defence mechanism when a cell is injured by engulfing portions of cytoplasm [12, 13]. The SD condition is relatively easy to apply in cell cultures and it is able to induce a condition of oxidative stress which has been used by numerous authors to mimic the ischemic environment [14–16].

At present, HT22 cells are still considered a good model for studying the neuronal cell death *in vitro* [17]. Steiger-Barraissoul [18] demonstrated that in serum deprived HT22 cells, there is cross-talk between apoptosis and autophagy, suggesting that autophagy is protective in these conditions. Indeed, the inhibition of autophagy by specific inhibitors enhances susceptibility to proapoptotic signals induced by SD. However, selective types of autophagy exist. The clearance of mitochondria by autophagy is known as mitophagy which appears to be an important tool in the control of mitochondria quality. Furthermore, several authors suggest that DRP1 is involved in mitophagy [19]. Indeed, the fission generates small mitochondria, which, due to their size, are efficiently engulfed by autophagosomes.

Melatonin is an ancient molecule found in the earliest unicellular organisms on earth. Initially identified as a secretory product of the pineal gland in mammals and other species, it was thought to be a hormone related to reproduction. The best-known actions of melatonin, supported by several studies [20–23], include antioxidant and antinflammatory properties [24]. Moreover, not only melatonin but also its secondary, tertiary and quaternary metabolites have proven to be powerful antioxidant and free radical scavengers as part of a cascade reaction known as melatonin’s antioxidant cascade [25]. As a broad spectrum antioxidant, melatonin has pleiotropic effects as well as neuroprotective properties.

In light of its particular characteristics, the present study was designed above all to investigate the role of melatonin in the mitochondrial network and whether it has beneficial effects on mitochondria reducing the autophagy and more specifically mitophagy.

In their outer membrane mitochondria express an integral protein called voltage-dependent anion channel (VDAC), which is involved in ATP/ADP exchange and metabolic trafficking across mitochondrial membranes [26]; this protein also plays a role in the apoptotic process by increasing mitochondrial membrane permeability. There is a growing body of evidence showing that VDAC can also be expressed in the plasma membrane (plVDAC) of different cell types, including neurons [27], in which it has several biologically relevant functions. Indeed, this channel is highly conductive for chloride, and therefore it can modulate the resting membrane potential and play a role in anion efflux and cell volume regulation [28]. Moreover, it can act as NADH-ferricyanide reductase [29]. On the other hand, this channel was found to be significantly activated during staurosporine-induced apoptosis in the mouse hippocampal cell line HT22 [8, 30], and its blockage was shown to prevent apoptosis, thus pointing to the crucial role of plVDAC in the apoptotic process. In view of these findings, the present study also sought to determine if plVDACs are involved in the apoptotic/autophagic process induced in HT22 cells by SD and whether the effects of melatonin on the mitochondrial network could result in changes in plasma membrane VDAC activity.

## Materials and methods

### Cell culture and treatment protocols

Hippocampal HT22 cells were maintained in DMEM-HAMS F12, supplemented with 10% fetal calf serum, L-glutamine (100mM) and 1% antibiotics (penicillin, streptomycin) and incubated in humidified 5% CO_2_ atmosphere at 37°C. At 80% confluence, cells were detached with trypsin-EDTA, washed and sub-cultivated in new flasks for 1–2 days before the experiments. The cells were then incubated at 37°C with two different concentrations of melatonin (200 and 500 μM) for 24 h before the exposure to SD. Ethanol was used as a vehicle for the melatonin and the final concentration did not exceed 0.1% (v/v).

### Clonogenic test

In order to explore potential cytotoxic effects of SD and the ability of melatonin to delay these effects we investigated the clonogenic survival of HT22 by counting the number of colonies. HT22 cells were grown in DMEM and 5x10^2^/ml cells were plated in six-multiwell dishes. Cells were incubated at 37°C to allow their adhesion on the dish, pre-treated with melatonin and then serum deprived for 24 h. Cells were fixed and stained by using a solution of ethanol and methylene blue in order to detect colonies. Colonies containing at least 50 cells were counted under the microscope. The experiment was performed in triplicate [31].

### Flow cytometric detection of cell death

To identify and evaluate the number of apoptotic cells in all experimental conditions, cells were stained using fluorescein isothiocyanate-conjugated Annexin V (FITC Annex-V; Immunostep) kit. Briefly, HT22 cells were resuspended in binding buffer (1x) and stained with 5 μl of Annex-V FITC according to the manufacturer’s instructions. Cells were then washed with phosphate-buffered saline (PBS), stained with propidium iodide (PI) and analyzed by flow cytometry (FC). Apoptotic cells were identified as Annex-V^+^ population, whereas Annex-V and PI staining represent the late apoptotic and necrotic cells.

Cytometric experiments were carried out with a FACSCanto II flow cytometer (BD Biosciences) equipped with blue (488 nm, air-cooled, 20 mW solid state), red (633 nm, 17 mW HeNe), and violet (405 nm, 30 mW solid state) lasers. Analyses were performed with FACSDiva^TM^ software (BD Biosciences). Samples were acquired by flow cytometry, collecting at least 10,000 events for each experimental condition [32].

### Detection of ROS production

The production of reactive oxygen species (ROS), mainly hydrogen peroxide (H_2_O_2_), was detected by CM-H_2_DCFDA (DCFDA, Molecular Probes). The fluorescent probe 5-(and-6)-chloromethyl-2,7’-dichlorodihydrofluorescein diacetate acetyl ester (CM-H_2_DCFDA) passively diffuses across the plasma membrane and is cleaved by intracellular esterases. The shift of fluorescence intensity is proportional to the amount of ROS produced within the cells. In all experimental conditions, cells were stained for 30 min at 37°C with 5μM CM-H_2_DCFDA (f.c). After the staining, cells were harvested by trypsinization and washed with PBS 3% FCS and then resuspended in 300 μl PBS for the cytometric analysis.

### Mitochondrial analyses

#### Cytometric assay for mitochondrial functionality

Mitochondria were studied by fluorescence labelling with MitoTracker Red CM-H_2_XRos (MTRC) a mitochondria-selective dye that accumulates in these organelles depending on the membrane potential [33]. 5 x 10^5^ /ml cells were directly labelled in six well plates at 37°C in 5% CO_2_ with 100 nM MTRC. After 30 min of incubation, cells were trypsinized, washed in PBS and subjected to flow cytometric analysis.

#### Mitochondrial morphology assessment

For confocal live imaging, cells were seeded on MatTek glass bottom chambers (MatTek Corporation) and stained with MTRC 200 nM f.c. for 30 min at 37°C in 5% CO_2._ Images were acquired by a Leica TCS SP5 II confocal microscope (Leica Microsystem, Germany) with 488, 543 and 633 nm illumination and oil-immersed objectives and averaged in real time using a line average of 32 to reduce random noise. The images were further processed and analyzed using ImageJ software (National Institutes of Health, Bethesda, MD). Raw images were binarized and mitochondrial morphological characteristics were quantified (area and perimeter of mitochondrion). NIH-developed ImageJ software was used for particle analysis. Mitochondria in the binarized confocal image were manually selected and individual particles (mitochondria) were analyzed for Area (A_m_) and Perimeter (P_m_). Form factor (FF: P_m_^2^/4πA_m_) was calculated from these values. FF has a minimal value of 1 when a particle is a small perfect circle and increase as the shape becomes elongated. An increase in FF represents an increase in particle length and complexity of the shape [34, 35].

### Autophagy detection

LC3 is a widely used marker of autophagosomes in mammalian cells. GFP-LC3 positive puncta were evaluated as previously described [28] using the Premo Autophagy Sensor Kit (Molecular Probes). Briefly, HT22 cells were transduced with BacMam LC3B-GFP with a MOI equal to 30, using 1×10^5^ cells in glass bottom chambers (MatTek Corporation). Mutated LC3B (G120A)-GFP was used as a negative control whereas chloroquine treatment was used as a positive control. Twenty-four hours after transduction, cells were treated as previously described. GFP-LC3B aggregates were observed using a confocal microscope and quantified by ImageJ software selecting the cells in the images and calculating the mean fluorescence intensity of all the pixels selected.

Autophagic vacuoles, mainly autophagolysosomes, were labeled with monodansylcadaverine (MDC; Sigma-Aldrich) by incubating cells grown on six-well plates with 0.05 mM MDC in PBS at 37°C for 10 min. After incubation, cells were trypsinized, washed two times in PBS and immediately analyzed by flow cytometry.

### Monitoring of mitochondrial autophagy (mitophagy)

In order to visualize mitochondrial engulfment in autophagic vacuoles we performed a co-labeling with LysoTracker Green (LTG) and MTRC. For confocal live imaging, cells were grown on MatTek glass bottom chambers and stained with LTG 200nM for 45 minutes at 37°C. During the last 15 min of incubation, cells were exposed to MTRC 200nM and then analyzed after 30 min using a Leica TCS SP5 II confocal microscope. Colocalization analyses were performed using JACoP plugin in ImageJ [36]. Pearson's correlation coefficient (PCC) was used as the parameter to measure co-localization in our samples.

The mitophagic flux was quantified by flow cytometry. Briefly, HT22 cells were stained with 50nM Mitotracker Deep Red (MTDR; Molecular Probes) for 15 min at 37°C and treated for 1h with 30 μM of the lysosomal inhibitor chloroquine (CQ, Molecular Probes) [37]. The MTRD MFI was evaluated before and after the treatment with CQ, and mitophagic flux was quantified as the ratio of the MTDR fluorescence w/o CQ.

### Measurement of oxygen consumption

The cells were washed once in glucose-free saline A (8.182 g/1 NaCl, 0.372 g/1 KC1 and 0.336 g/1 NaHCO_3_) and resuspended in the same buffer at a density of 1×10^7^ cells/ml. Oxygen consumption was measured using a YSI oxygraph equipped with a Clark-type electrode (model 5300, Yellow Springs Instruments Co., Yellow Springs, OH, USA) at 37°C. The cell suspension (3 ml) was transferred to the polarographic cell and, under constant stirring, basal respiration was measured immediately after the addition of 5 mM glucose over a 3-min period. Five mM pyruvate was then added and the rate of oxygen utilization was monitored for a further 3 min. The rate of oxygen utilization was calculated as described in ref. [38].

### Electrophysiological recordings

HT22 murine hippocampal neuronal cells were cultured on sterilized coverslips placed in wells. Four experimental conditions were considered: 1. control; 2. melatonin; 3. SD; 4. SD induced in cells pre-loaded with melatonin. Melatonin pre-loading was carried out as described above and experiments were performed 24h after the beginning of SD.

Electrophysiological recordings were carried out using patch clamps in cell-attached and outside-out modes for investigating ion channel currents mediated by voltage-dependent anion channels in plasma membrane (plVDACs). The experiments were performed under visual guidance using a Zeiss Axioskop microscope (Carl Zeiss International, Italy) equipped with an infrared videocamera connected to a monitor; recordings were carried out using an Axopatch-200B amplifier (Axon Instruments, USA). Each coverslip was transferred into a recording chamber, where the cells were superfused throughout recordings with the extracellular solution at a rate of 3 ml/min. The extracellular solution contained in millimolar: 140 NaCl, 5 KCl, 10 HEPES, 1 MgCl_2_, 1.8 CaCl_2_, 23 sucrose (pH 7.4). Recording electrodes were pulled from borosilicate glass tubes (World Precision Instruments, USA) using a vertical puller (model PP-830 Narishige, Japan) and had 3–5 MΩ tip resistance when filled with the extracellular solution used for cell- attached recordings. Holding voltage (V_H_), defined as bath voltage-electrode voltage, was 0 mV; currents were recorded for voltage steps from +100 to -100 mV separated by 20 mV. Following each cell-attached recording, the electrode was gently withdrawn to switch to outside-out configuration, in which the external and internal membrane lipid layers were bathed with the extracellular solution; the same voltage steps were applied to record plVDAC currents. Data analysis was performed offline by WinWCP software (Strathclyde electrophysiology software analysis V 3.2.9, John Dempster, University of Strathclyde, UK).

### Sub-cellular fractionation and western blotting

The cells were processed to obtain the mitochondrial and cytosolic fractions, as described in Cantoni et al [39]. Equal amounts (25 μg) of the mitochondrial and cytosolic fractions were resolved in sodium dodecyl sulfate polyacrylamide gel and electrotransferred to polyvinylidene difluoride membranes. Western blot analyses were performed using antibodies against DRP1, HSP-60 and actin (Santa Cruz, CA).

For protein extraction and subsequent analysis, HT22 cells were grown at a density of 7 x 10^4^ cells per well in 6 well plates. After treatments, the cells were processed to obtain the mitochondrial and cytosolic fractions, as described in Cantoni et al. [39], or whole cell lysate as reported below and then processed for western blot analyses. Briefly, to obtain the whole cell lysate, HT22 cells were lysed with 50–100 μl of RIPA buffer (150 mM sodium chloride, 50 mM Tris pH 8.0, 1.0% NP-40, 0.5% sodium deoxycholate, 0.1% SDS) supplemented with 10 mM sodium fluoride, 1mM sodium orthovanadate and 1 tablet per 50 ml of SIGMA*FAST* Protease Inhibitor (Sigma- Aldrich, Italy). After centrifugation at 15,000 x g for 15 min at 4°C, the supernatants were stored at -80°C until further use. Protein amounts were determined with the Bio-Rad Protein assay (Bio-Rad, Italy). Absorption at 595 nm was determined using a DU 640B spectrophotometer (Beckman Coulter, Italy) and protein amounts of the test samples were calculated from the standard curve. For SDS-PAGE, samples containing 30 μg of protein were mixed with Laemmli sample buffer and loaded onto 10% and/or 12% SDS-PAGE gels. Subsequently, proteins were blotted to a PVDF membrane (Thermo, Italy). The primary antibodies used were: anti-phospho-DRP1 (Ser 637; #4867 Cell Signaling; 1:1000 dilution), anti-DRP1 (Santa Cruz Biotechnology Santa Cruz, CA; 1:300 dilution) anti-Cytochrome *c* (Santa Cruz Biotechnology; 1:1000 dilution), anti-Bcl-2 (Santa Cruz Biotechnology; 1:1000 dilution), anti-Bax (Santa Cruz Biotechnology; 1:1000 dilution), anti-p62 (Sigma-Aldrich, Italy; 1:2000 dilution), anti-HSP60 (Santa Cruz Biotechnology; 1:1000 dilution) and anti-Actin (Sigma-Aldrich, Italy; 1:2000 dilution). Primary antibodies were incubated overnight at 4°C, followed by washing and incubation with secondary HRP-conjugated antibody (Jackson Immunoresearch). Immune complexes were visualized using the ECL FAST PICO reagent (Immunological Sciences). Antibodies against actin and HSP-60 were used to assess the purity of the fractions and the equal loading of the lanes. Densitometric analyses of the bands were performed using ImageJ software (NIH, Bethesda, MD).

### Statistical analysis

Data are shown as mean ± standard deviation (s.d.) of at least three independent experiments. Differences between groups were analyzed using a one-way analysis of variance (One-way ANOVA), followed by a Bonferroni *post hoc* analysis or a Dunnett’s test. P values less than 0.05 were considered statistically significant. All statistical analyses were performed using GraphPad Prism 5.0 (GraphPad software).

## Results

### Detection of cell death and proliferation

**Fig 1:**
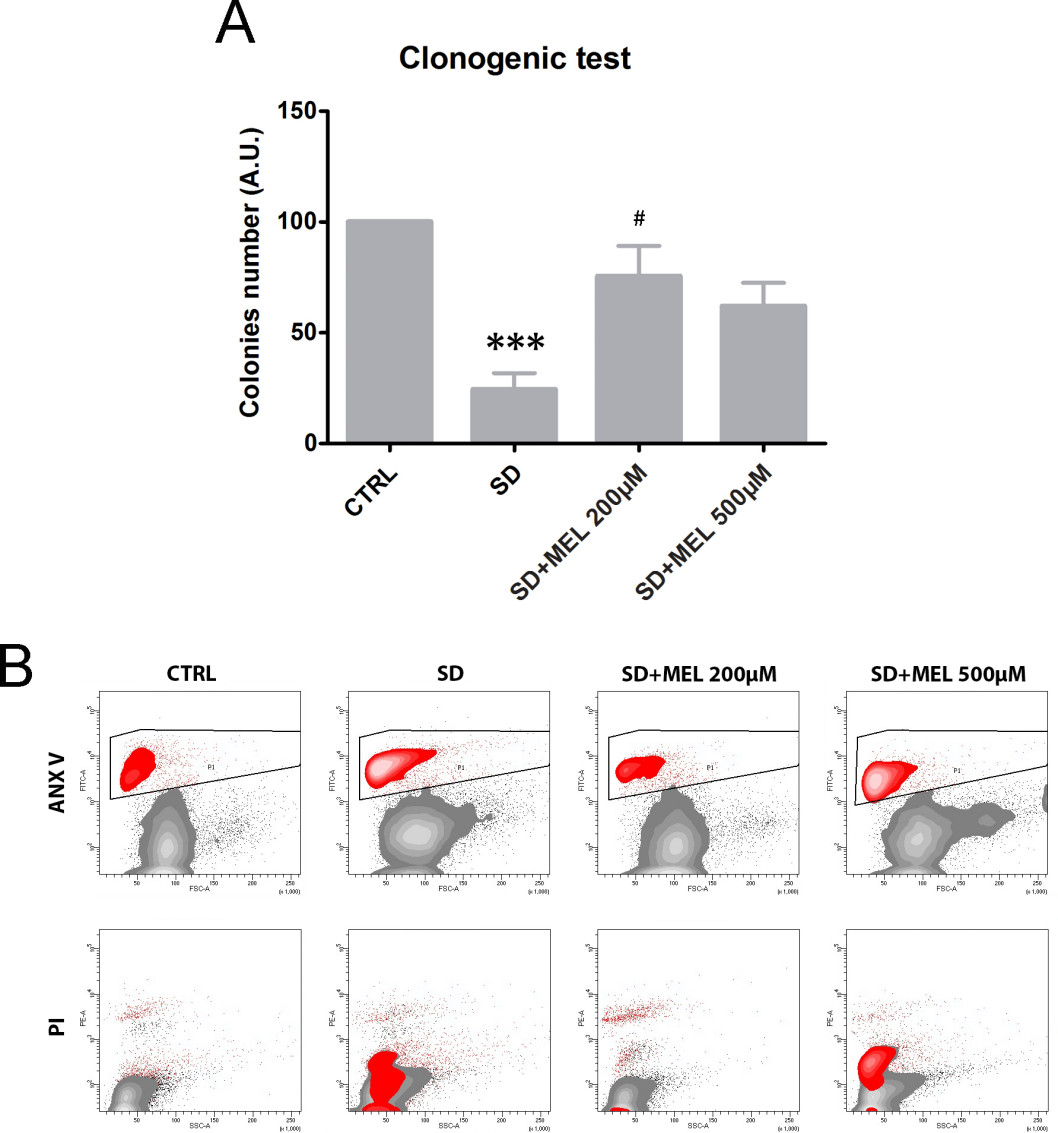
Evaluation of cell death and proliferation. (**A**) Colonies were stained and the surviving fraction was determined in all experimental conditions: untreated cells (CTRL), 24h serum deprived cells, melatonin (MEL) 200 μM and melatonin 500 μM pre-loaded starved cells. Each value is expressed as an absolute number ± s.d. (results from n = 3 independent experiments); ***P < 0.001 *vs* control, ^#^P < 0.05 *vs* serum deprivation (SD). (**B**) Contour plots of a representative experiment illustrating Annex-V (upper row) and PI positivity (bottom row) of HT22 cells. Of note, Annex-V positive cells were negative for PI fluorescence highlighting an apoptotic pathway.

To examine the role of melatonin in neuronal cell damage induced by SD we pre-treated HT22 cells for 24h with melatonin, and to determine the effects of combining SD with melatonin we assessed the clonogenic survival of HT22 cells. The clonogenic assay showed a significant (P < 0.001) cell killing of HT22 cells challenged with SD for 24h (Fig 1A). The melatonin pre-loading was able to restore the cell viability of starved cells to values similar to those of controls and this effect was statistically significant (P < 0.05) for the 200μM concentration. Indeed, the clonogenic assay showed 70% cell killing of HT22 cells exposed to SD, whereas the pre-loading with both concentrations noticeably reduced cell killing by about 35%. To evaluate the apoptotic stage of cells following treatment with SD in the absence and the presence of melatonin we analyzed Annex-V/PI positivity in all experimental conditions. Fig 1B shows representative cytograms for each treatment assayed. SD induced an increase in the percentage of apoptotic (Annex-V^+^) and necrotic (PI^+^) cells. When the cells were challenged with both melatonin doses for 24h, the percentage of Annex-V^+^ and PI^+^ cells decreased. The Annex-V^+^ cells are shown within the designated area in the upper portion of the first row of cytograms, whereas the PI^+^ positive cells are highlighted in the lower portion of the cytograms in the second row. The protective effect of melatonin was particularly evident at 200μM which was consistent with the results of the clonogenic assay.

### Analyses of mitochondrial features and functions

**Fig 2:**
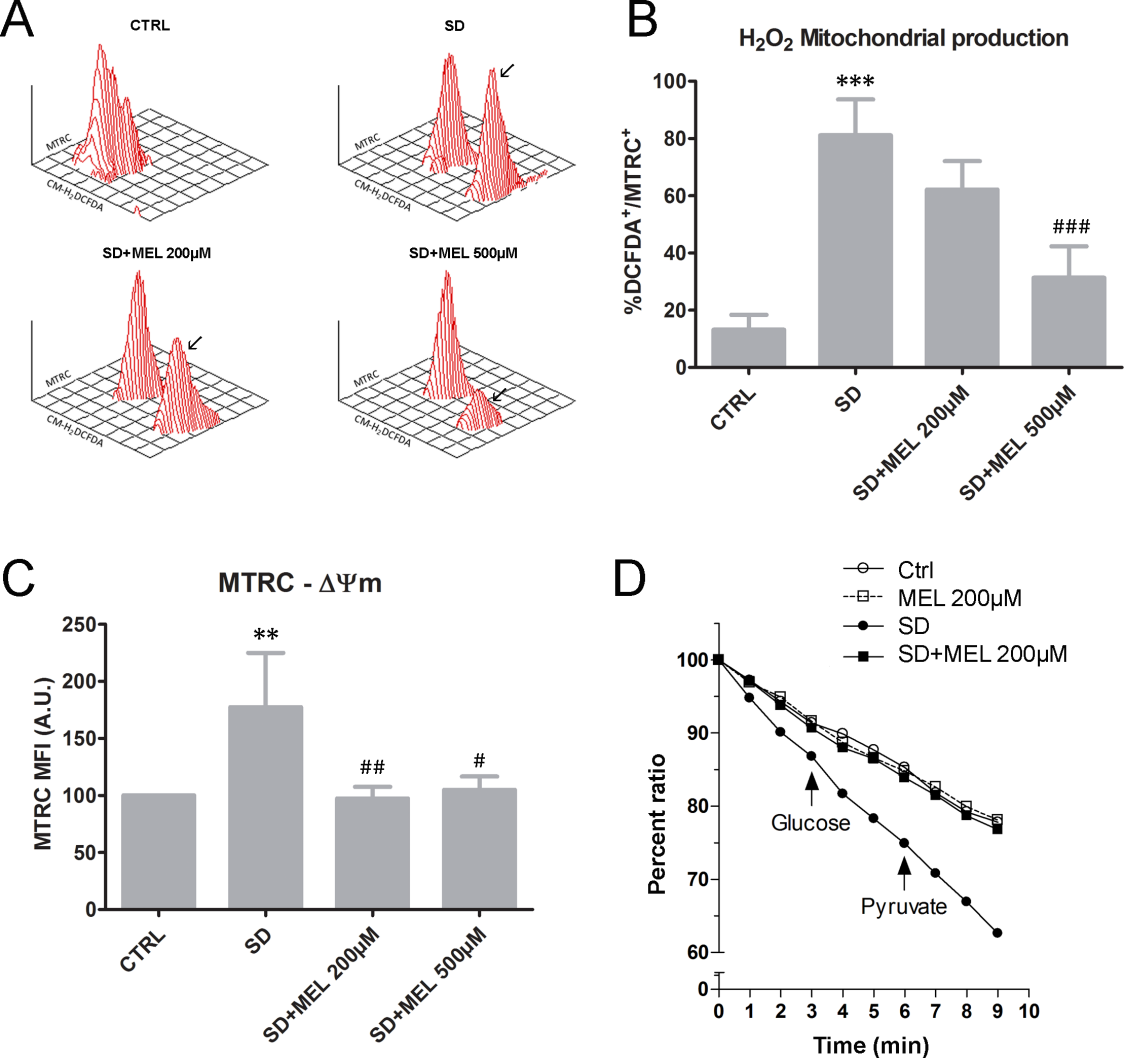
Evaluation of mitochondrial features. (**A**) 3D plots MTRC *vs* CM-H_2_DCFDA (DCFDA) for all experimental conditions. The DCFDA^+^/MTRC^+^ population, absent in the control condition, is particularly evident in SD samples, whereas it appears to a lesser extent in MEL pre-loaded samples. (**B**) Statistical histogram related to the DCFDA^+^/MTRC^+^ percentage in HT22 cells for each experimental condition. Each value is expressed as a percentage ± s.d. (n = 3); ***P < 0.001 *vs* control, ^###^P < 0.001 *vs* SD. (**C**) Statistical histogram related to the MTRC MFI shift in HT22 cells for each experimental condition. Mean values were converted to arbitrary units (A.U.) setting control cells as 100. Each value is expressed as a mean ± s.d. (n ≥ 3 independent experiments); **P < 0.01 *vs* control, ^#^P < 0.05 and ^##^P < 0.01 *vs* SD. (**D**) Basal respiration in control (25.96 ± 0.68 nmol O_2_/min/10^7^ cells) or in SD (38.24 ± 0.55 nmol O_2_/min/10^7^ cells) cells was recorded for 3 min in the presence of 5 mM glucose. Oxygen consumption was then monitored for a further 3 min after the addition of 5 mM pyruvate. Data are expressed as the percent ratios of the oxygen concentration measured at each time point to time 0.

Oxidative stress is generally induced by SD. Furthermore, a growing body of evidence points to a close interaction between redox status and mitochondrial dynamics, although at present, it is uncertain whether mitochondrial fission induces oxidative stress or oxidative stress disrupts mitochondrial dynamics [10]. To examine the effect of melatonin on SD-induced oxidative stress we measured the change in ROS levels within mitochondria stained with MTRC by flow cytometry. We found that SD treatment promotes the formation of two populations corresponding to the Gaussian slopes if compared to the controls, one corresponding to the DCFDA-/MTRC^+^ population, and another corresponding to the DCFDA^+^/MTRC^+^ population. In particular, the latter population represents approximately 80% of the mitochondria in serum deprived cells. We also found that melatonin pre-loading inhibited the percentage of DCFDA^+^/MTRC^+^ events. Indeed, this percentage drops to 60% and 30% for melatonin concentrations of 200 and 500 μM, respectively. Only the concentration of 500 μM caused a significant reduction of DCFDA^+^/MTRC^+^ events (P < 0.001). These results show that melatonin reduces the number of mitochondria with a high ROS content (Fig 2A and 2B).

Previous studies have shown that mitochondrial membrane hyperpolarization is associated with ROS production [40]. MTRC is a dye that stains mitochondria in live cells and its accumulation is dependent upon membrane potential. We used MTRC to measure mitochondrial membrane potential (Fig 2C). The statistical histogram on MTRC behaviour showed that SD elevated MTRC MFI 2.23 fold compared to that of the controls after 24h of exposure (P < 0.01). Furthermore, it is noteworthy that 200 and 500 μM melatonin pre-treatment brought about a reduction in the MTRC positivity in HT22 cells (P < 0.01 and P < 0.05, respectively).

Respiration is thought to drive proton translocation across the inner mitochondrial membrane. In order to test this hypothesis we performed oxygen consumption experiments. The results revealed that SD treatment significantly increased basal cellular respiration in HT22 cells (38.24 ± 0.55 nmol O_2_/min/10^7^ cells) compared to control conditions (25.96 ± 0.68 nmol O_2_/min/10^7^ cells). Indeed, the extent of oxygen utilization in response to glucose (5 mM) or pyruvate (5 mM) was markedly increased in SD cells (Fig 2D). Interestingly, combining SD with 200μM melatonin pre-loading normalized oxygen consumption. In light of this result, we did not apply 500μM melatonin. In addition, melatonin pre-loading did not alter oxygen consumption in control cells. Taken together these results show that SD increases mitochondrial respiration, leading to an increase in mitochondrial membrane potential, whereas melatonin prevents these effects.

**Fig 3:**
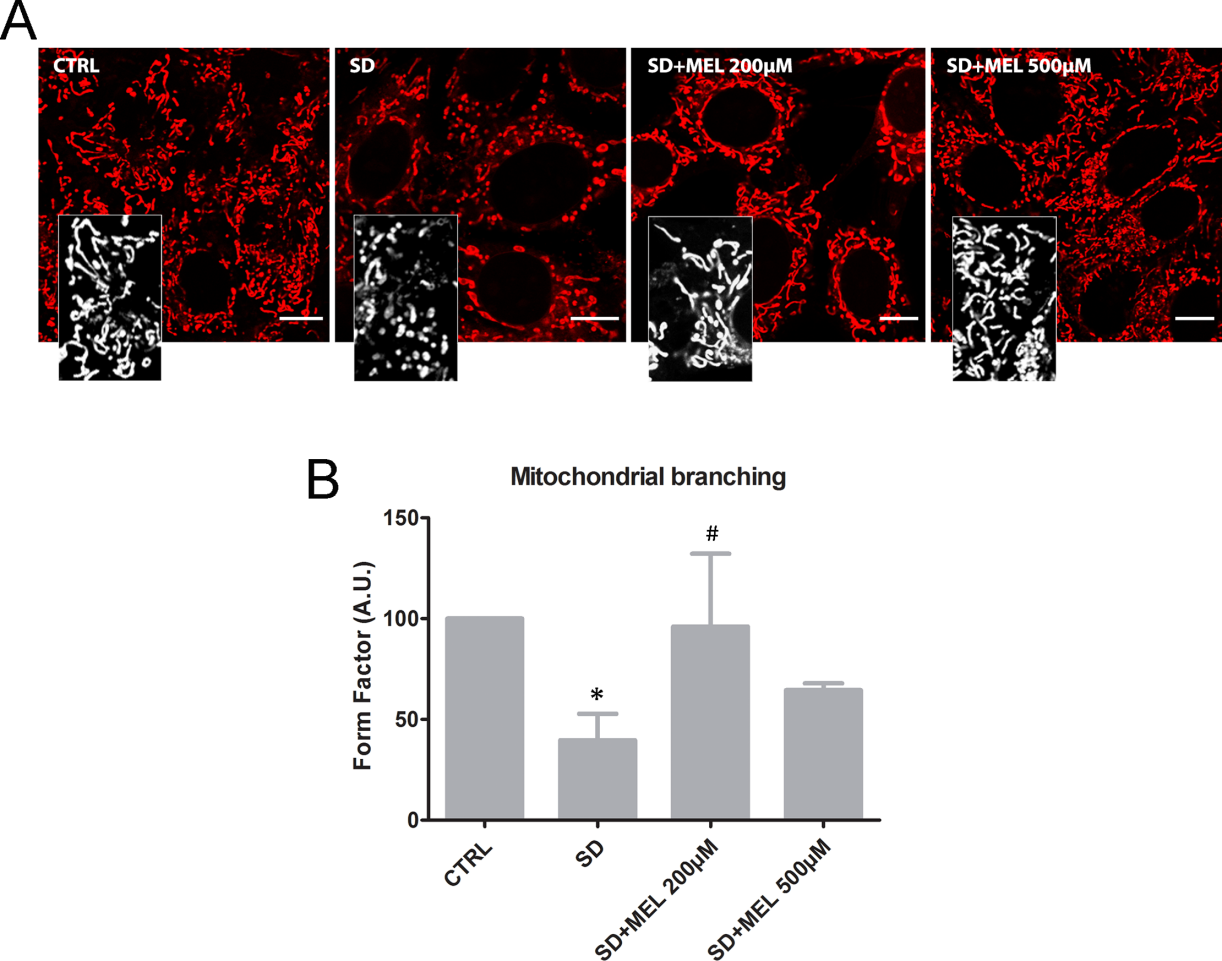
Assessment of mitochondrial morphology. (**A**) Representative confocal images of mitochondrial morphology in HT22 cells labelled with MitoTracker Red CM-H_2_XRos. Bars: 10 μm. Inset: detail of mitochondria (**B**) Quantification of mitochondrial branching (Form Factor). FF values were converted to arbitrary units (A.U.) setting control cells as 100 (n ≥ 3 independent experiments; ≥10 images assessed per experiment); *P < 0.05 *vs* CTRL, ^#^P < 0.05 *vs* SD.

To further characterize the role of SD in mitochondrial fragmentation and the preservation of mitochondrial structure by melatonin pre-loading, we analyzed mitochondria morphology by MTRC. Cell images captured using confocal microscopy revealed two types of mitochondrial morphologies; tubular and fragmented. The tubular structure in mitochondria was prominent in control and melatonin pre-treated cells, whereas SD exposure severely reduced mitochondrial size and disrupted mitochondrial reticularity, shifting the tubular structure towards a round shape (Fig 3A). The form factor (FF) values were used as mitochondrial shape descriptors to estimate mitochondrial fission and to quantify the length and degree of branching in mitochondria. As shown in Fig 3B, the SD treatment induced a 50% reduction (P < 0.05) in mitochondrial branching, whereas the 200μM melatonin pre-loading restored the degree of mitochondrial branching to those found in controls (P < 0.05). In the cell mitochondria exist in an every-changing dynamic state, where mitochondrial networks are constantly elongating and dividing. The balance between these two events provides an equilibrium of small fragmented mitochondria and long interconnected mitochondrial networks and is thought to be essential for mitochondrial homeostasis which plays a key role in cell survival and stability [14, 41].

**Fig 4:**
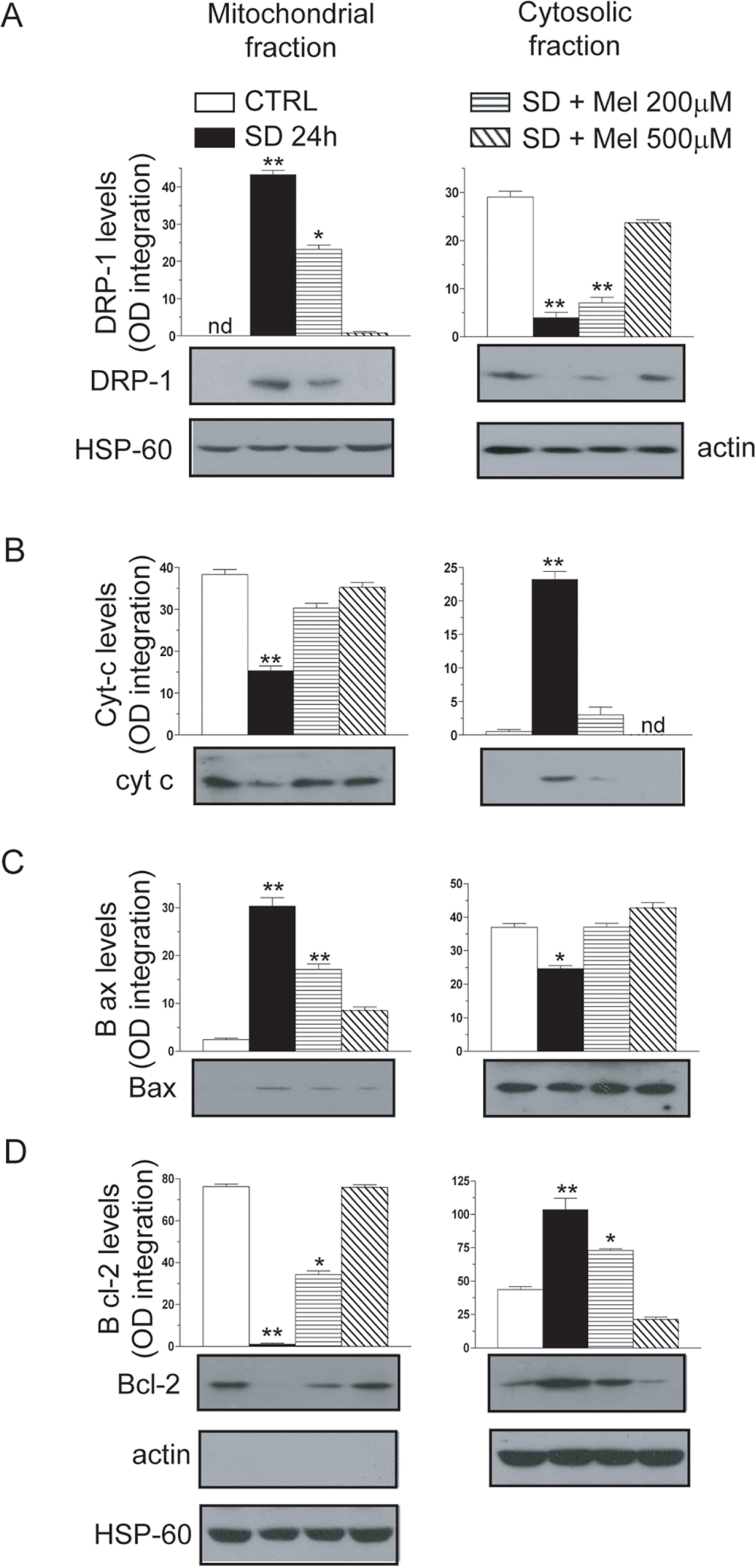
DRP-1 mitochondrial accumulation induced by 24h serum deprivation is sensitive to melatonin pre-exposure and associated with cytochrome *c* mitochondrial release. Cells were incubated with two different concentrations of MEL (200 and 500 μM) for 24 h before SD. The cells were then processed to obtain the mitochondrial and cytosolic fractions analysed by western blot to determine DRP-1, cytochrome *c*, Bcl-2 and Bax sub-cellular localization. Blots shown in B, C and D were obtained re-probing the same membrane for the proteins indicated using the appropriate antibodies. Blots shown were also probed for actin and HSP-60 and are representative of three separate experiments with similar outcomes. The relative amount of DRP1, cytochrome *c*, Bcl-2 and Bax proteins was quantified by densitometric analysis (nd, not detectable). Results represent the means ± s.d. calculated from 3–5 separate experiments. *P < 0.05, **P < 0.01 compared to untreated cells (one-way ANOVA followed by Dunnett’s test).

To further explore the mechanism through which mitochondrial dynamics were impaired during SD treatment we assessed the expression of p-DRP1 and DRP1 with or without melatonin in HT22 cells. We investigated the recruitment of p-DRP1 to the mitochondria by measuring the protein levels of DRP1 in isolated mitochondria and cytosol. DRP1 localized to mitochondria only in the SD and 200μM melatonin pre-treatment conditions, although the level of protein was lower in the sample pre-treated with 200μM melatonin. On the other hand, importantly, melatonin pre-loading completely prevented (500μM) or partially prevented (200μM) the translocation of DRP1 to mitochondria (Fig 4A).

Because mitochondrial fragmentation was found either with or without apoptotic features, we investigated the release of cytochrome *c* (cyt-*c*), a typical hallmark of apoptosis. We found that cyt-*c* was higher in the cytosolic fraction only in the SD sample, whereas the melatonin pre-loading completely prevented this increase (Fig 4B). These results imply that mitochondrial fragmentation occurs concurrently with apoptosis as shown in panels C and D. The preferential mitochondrial localization of DRP1 is also associated with Bax translocation to the organelle and the loss of mitochondrial content of Bcl-2, pro- and anti-apoptotic proteins, respectively. The effects mediated by SD are completely prevented by melatonin pre-loading. Concurrently, SD treatment resulted in the dephosphorylation and activation of pDRP1, which was inhibited by 200μM melatonin and partially prevented by 500μM melatonin (S1 Fig).

### Assessment of autophagy and mitophagy

**Fig 5:**
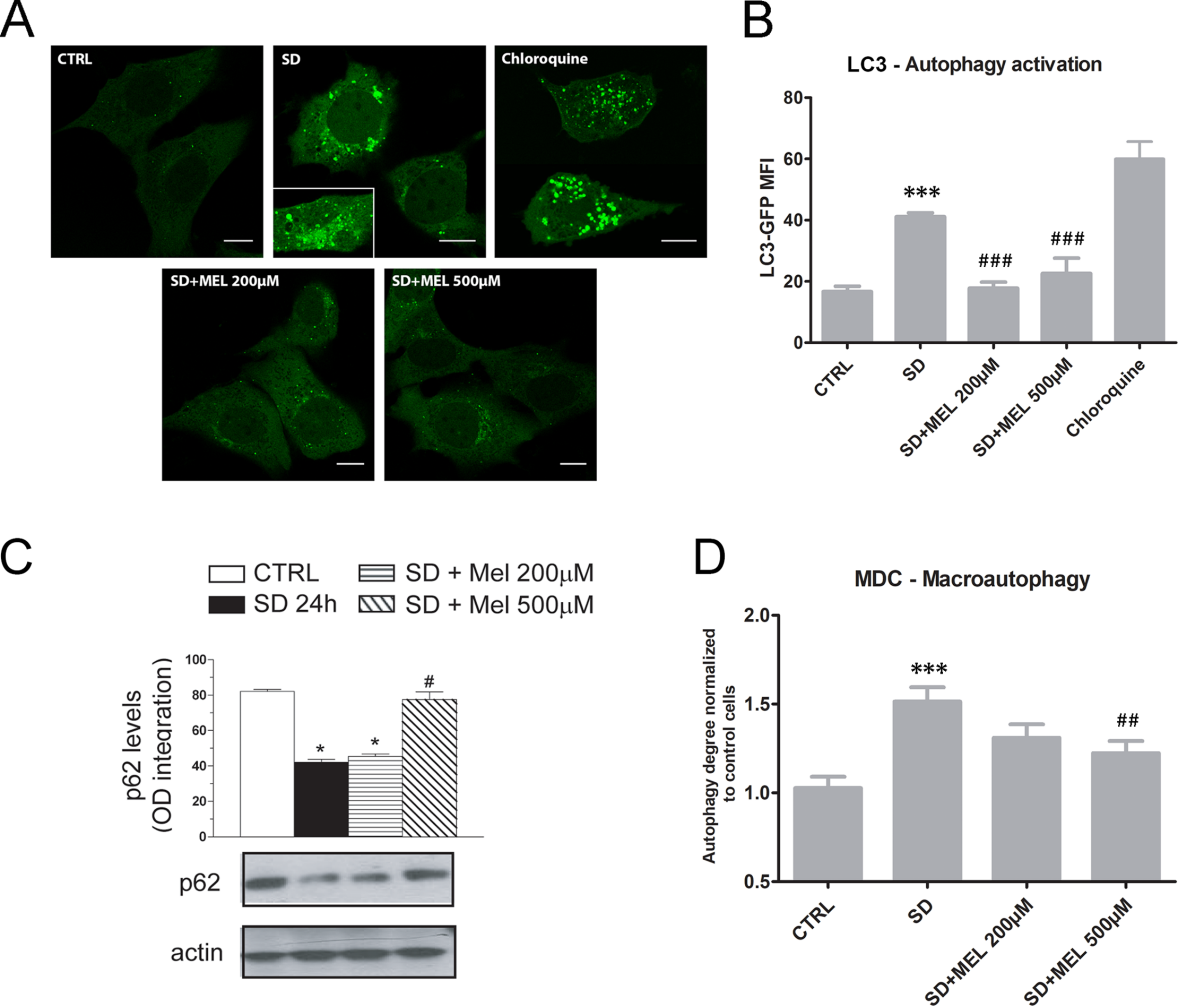
Autophagy detection by LC3B-GFP, MDC and p62. (**A**) Single confocal optical sections (~0.8 μm thickness) showing LC3B-GFP positive puncta in HT22 cells. Bars: 10 μm. (**B**) Statistical histogram depicting MFI variation of LC3B-GFP in HT22 cells obtained from confocal microscopy images by ImageJ software. Each value is expressed as a mean ± s.d. (Results from n ≥ 3 independent experiments); ***P < 0.001 *vs* control; ^###^P < 0.001 *vs* SD. (**C**) 24h serum deprivation induces loss of p62 content sensitive to melatonin pre-exposure. Cells were incubated with two different concentrations of MEL (200 and 500 μM) for 24 h before SD. The cells were then processed to obtain the whole cell lysate and analysed by Western blot to determine p62 levels. Blot shown was also probed for actin and is representative of 3 separate experiments with similar outcomes. The relative amount of p62 protein was quantified by densitometric analysis. Results represent the means ± s.d. calculated from 3 separate experiments. *P < 0.05 *vs* CTRL, ^#^P < 0.05 *vs* SD (one-way ANOVA followed by Dunnett’s test). (**D**) Statistical histogram related to the MDC MFI shift in HT22 cells for each experimental condition. Mean values were converted to arbitrary units (A.U.) setting control cells as 1.0. Each value is expressed as a mean ± s.d. (n ≥ 3 independent experiments); ***P < 0.001 *vs* control, ^##^P < 0.01 *vs* SD.

**Fig 6:**
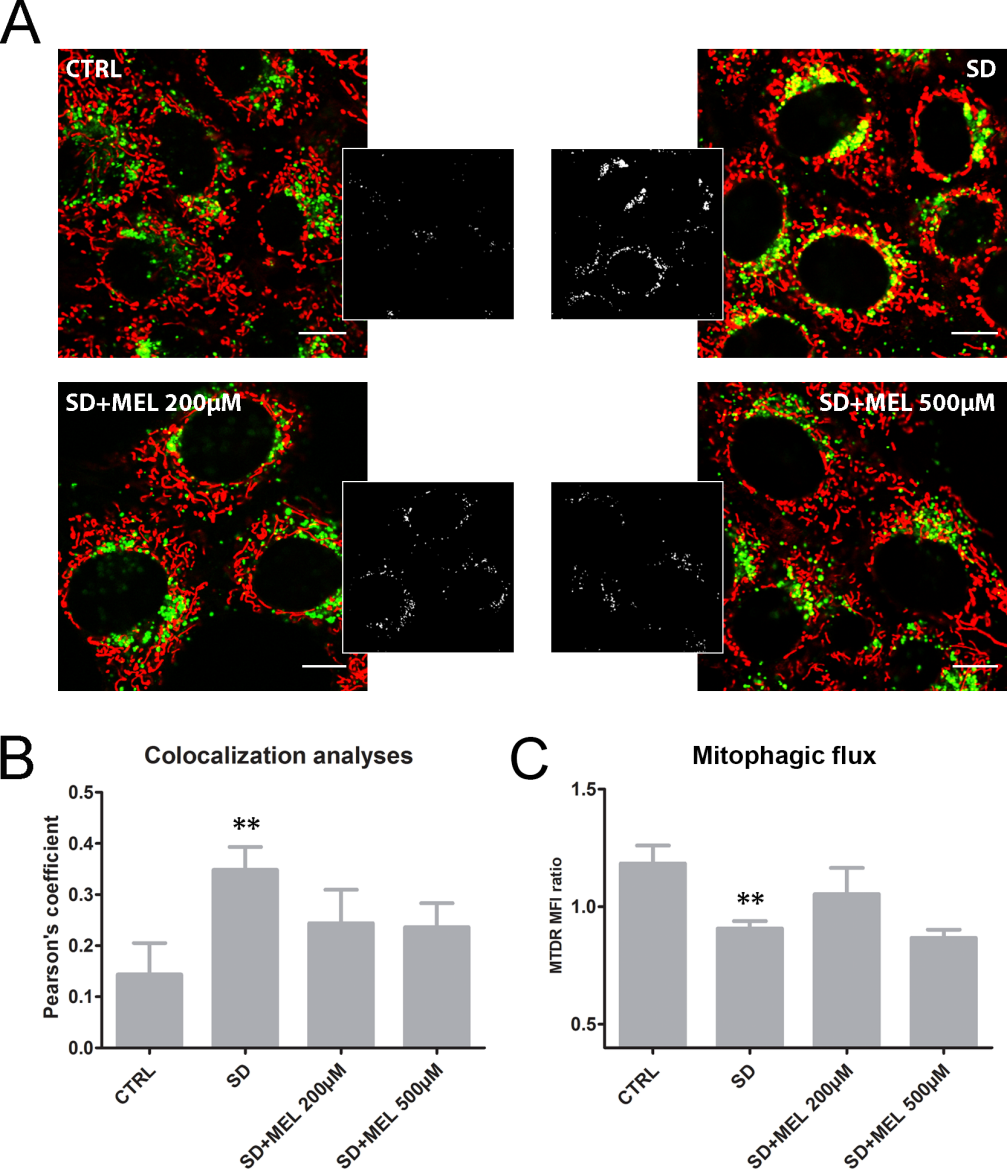
Assessment of mitochondrial autophagy. (**A**) Single confocal optical sections (~0.8 μm thickness) showing overlay of LTG (green) and MTRC (red) in HT22 cells. Bars: 10 μm. Inset: Binary versions of images shown in A, after applying threshold: the colocalization mask was generated by ImageJ software to show colocalization pixels. Pixels with positive signals for both probes are shown in white. The cells shown are representative of most of the analyzed cells. (**B**) Pearson's colocalization coefficient (PCC) of LTG and MTRC in HT22 cells. Pearson's coefficients were derived from three completely independent experiments with >10 fields per experiment contributing to the cumulative result. Each value is expressed as PCC ± s.d.; **P < 0.01 *vs* CTRL. (**C**) Mitophagic flux for each experimental condition. MTDR fluorescence was measured in the presence or absence of chloroquine (CQ). Each value is expressed as the ratio ± s.d. of MTDR MFI in the presence of CQ to that in the absence of CQ (n ≥ 3 independent experiments); **P <0.01 *vs* CTRL.

Mitochondria can be degraded by non-selective autophagy, as well as by mitophagy, a selective pattern of autophagy. Non-selective autophagy and mitophagy have been shown to be triggered in response to nutrient deprivation, starvation or rapamycin [42, 43]. To understand whether and how melatonin affects autophagy, we stably transfected HT22 cells with GFP-LC3B to investigate the formation of autophagosomes. LC3, the microtubule-associated protein 1A light chain 3, is normally located throughout the cytoplasm, but becomes concentrated in autophagosomes during autophagy. It is generally agreed that LC3 is the most reliable cellular marker for autophagy activation [44]. SD treated cells exhibited numerous bright LC3B-positive puncta (Fig 5A) and had increased MFI for LC3B (Fig 5A) compared to the control condition (P < 0.001). The number of LC3-positive puncta visibly decreased for both melatonin concentrations. Similarly, quantification of LC3 fluorescence (Fig 5B) showed a significant (P < 0.001) decrease in LC3 MFI in melatonin pre-loaded samples. P62 is a substrate degraded by autophagy and a reduction in its amount reflects an increase in the autophagic pathway [45]. SD-treatment induced a reduction in p62 level (P < 0.05) whereas 500 μM of melatonin restores this level to value similar to that of control (P < 0.05; Fig 5C). The macroautophagy was also evaluated by MDC. This dye has been indicated as a specific marker for autophagolysosomes [46]. Our results demonstrated that SD treatment induces a significant (P < 0.001) increase in MDC MFI. On the other hand, both melatonin concentrations reduce the autophagy as demonstrated in Fig 5D. Previous studies have shown that damaged mitochondria are removed mainly through the autophagic pathway in other conditions [47]. The quantity of mitochondria engulfed by lysosomes was measured using a double staining technique. Confocal images confirmed that SD exposure led to an increase in the size/number of the acidic lysosomal/autophagosomal compartment. Furthermore, green lysosomal fluorescence co-localized with red mitochondria fluorescence appearing yellow-orange in the overlaid areas of the red and green fluorescence channels. However, melatonin reduced the colocalization of red and green fluorescence (Fig 6A). This behaviour is quite evident in the colocalization mask in which pixels with positive signals for both probes are shown in white. Pearson’s correlation coefficient (PCC) was used as a standard to measure colocalization between the green fluorescence of LTG and the red fluorescence of MTRC. This coefficient was also used to detect mitophagy (Fig 6B). Autophagic vacuole/mitochondrial colocalization was particularly evident in serum-deprived cells (P < 0.01), with a Pearson’s correlation coefficient of about 0.35 ± s.d., whereas melatonin pre-loading induced a decrease in this value. The mitophagy is a selective type of autophagy able to eliminate unwanted or damaged mitochondria. The mitophagy was also assessed by mitophagic flux. The decrease in MTRD MFI in SD sample demonstrated an activation of mitophagy (P < 0.01) whereas the pre-loading with 200 μM of melatonin restores the mitophagic flux to value similar to that of control.

### Electrophysiological analyses of plVDAC channels

**Fig 7:**
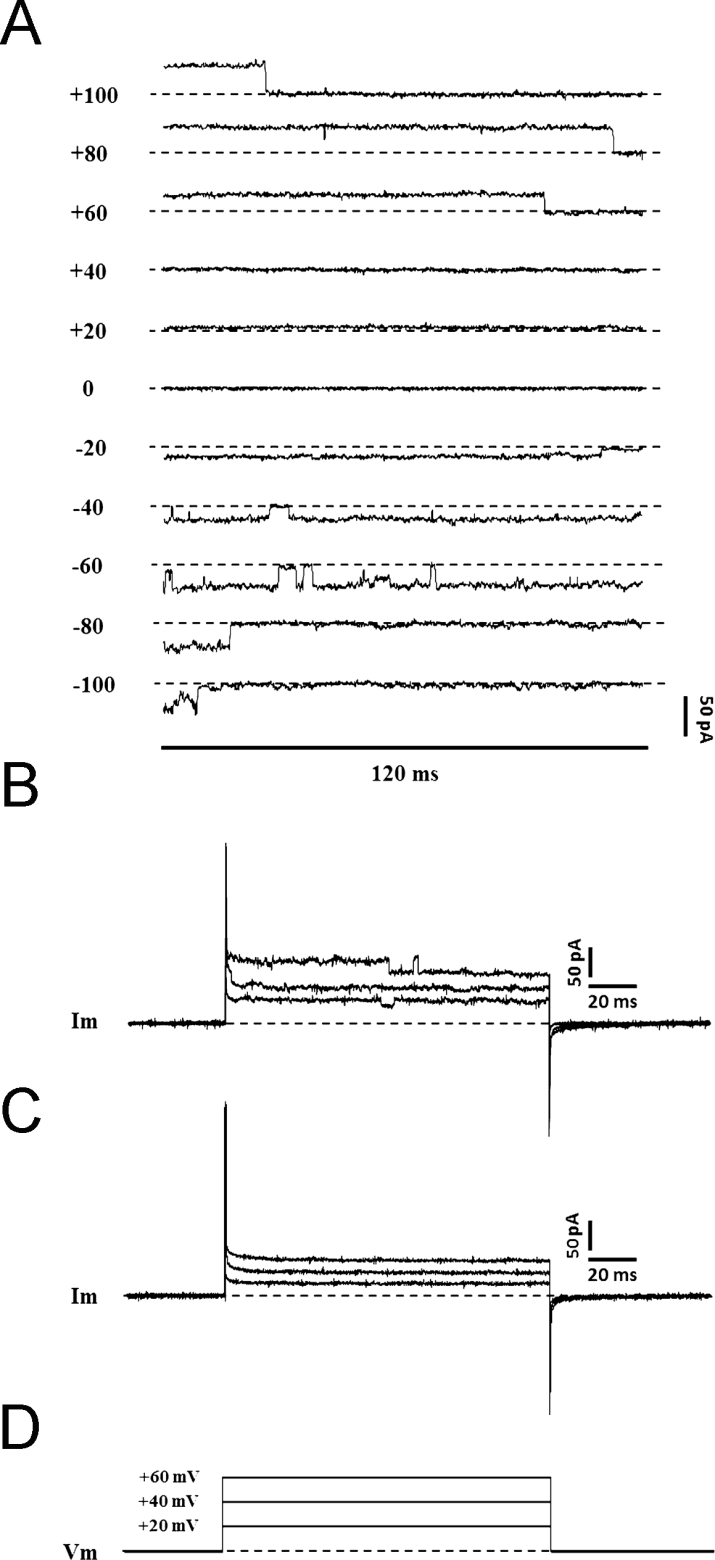
Electrophysiological recordings of voltage-dependent anion channels (VDACs) in the plasma membrane. Conductance of VDAC in response to a ramp of voltage steps from -100 mV to + 100 mV (separated by 20 mV) recorded in outside-out configuration of excised HT22 cell membrane patches exposed to pipette solution without ATP, starting from 0 mV holding voltage. The dashed lines refer to the closed state of the VDAC channel **(A)**. The presence of opened VDACs in plasma membrane of HT22 cells evaluated in cell-attached configuration: representative trace of a cell showing opened VDACs in HT22 cells after 24h of SD **(B)**, and trace of a cell showing closed VDACs in cell-attached configuration in HT22 cells after combining SD with MEL pre-loading **(C)**, in response to 20 mV, 40 mV and 60 mV depolarizing steps of 100 ms each **(D)**.

To gain insights into the possible involvement of plVDACs in serum deprivation-induced autophagy/apoptosis in HT22 cell line, we analyzed the activation state of these channels in our experimental conditions [30]. In agreement with the literature, we found that HT22 neuronal cells displayed a low electrical activity under control conditions in cell-attached configuration, without showing any currents compatible with voltage-dependent anion channel activation. We subsequently performed recordings in outside-out configuration from a high percentage of excised membrane patches exposed to the pipette solution without ATP, a simple mechanism for channel activation upon excision [25]. Under these recording conditions, we uncovered the presence of voltage-dependent anion channel-mediated currents (Fig 7A). These findings show that plVDACs are expressed in the plasma membrane of HT22 cells, but they are silent in control conditions of negative resting membrane potential, normal concentration of ATP and in the absence of any apoptotic signal. Conversely, SD was able to induce large square-like single-channel currents, similar to those frequently found in apoptotic cells, in 28.6% of HT22 neuronal cells recorded in the cell-attached mode (Fig 7B and 7D). Moreover, in outside-out patch clamp experiments, activation of these channels was found to be blocked by using 4 mM ATP in the pipette solution, pointing to the VDAC nature of these channels. On the other hand, when SD was combined with 200μM melatonin pre-loading, VDAC-like currents were not recordable in any HT22 neuronal cells, showing the protective effect exerted by melatonin against damage induced by starvation (Fig 7C and 7D). In light of this result, we did not apply 500μM melatonin. Taken together, our electrophysiological results show that: i) plVDACs are also involved in autophagy/apoptotic process induced by SD in HT22 cell line; ii) melatonin pre-loading was able to prevent plVDAC opening, thus possibly reducing their contribution to the autophagic/apoptotic process.

## Discussion

A growing body of evidence indicates that mitophagy is a mechanism able to maintain an appropriate mitochondrial network. However, although important studies have demonstrated that melatonin affects the translocation of Fis1 (mitochondrial fission 1 protein) and DRP-1 reducing mitochondrial fission, more studies are needed to explain the role of melatonin in the mitochondrial dynamics [48–50]. On the other hand, it is well known that melatonin has beneficial effects in several stress conditions by regulating oxidative stress and mitochondrial dysfunction. Numerous investigations have also shown that melatonin preserves mitochondrial homeostasis. Indeed, melatonin’s multiple contributions to the maintenance of mitochondrial homeostasis have made it an increasingly interesting pharmacological agent against neurodegenerative diseases and we direct readers to excellent papers on this topic [10, 35, 51]. Moreover, our previously published data have shown that melatonin reduces oxidative stress after various injuries affecting mitochondrial Δψ and cardiolipin content [52, 53]. In this regard, the present study was designed to investigate how melatonin affects SD-induced toxicity, focusing on the mitochondrial network.

Our results show that in hippocampal HT22 cells, SD induces a type of death that shares features of apoptosis and autophagy, concomitantly with the translocation of DRP1 from the cytoplasm to the mitochondrial fraction. Our findings are in agreement with Steiger-Barraissoul’s investigation [18], in which the authors suggest that in HT22 starved cells the autophagy mechanism seems to be more protective than deleterious. Our results show that that SD is able to induce neuronal death as demonstrated by both Annex-V/PI positivity and the release of cyt-*c* in the cytoplasm. In addition, our results demonstrate that melatonin is able to counteract these effects, although the molecule’s effects are not dose-dependent. These results appear to be in consistent with recent papers that have reported a pro-oxidant role of melatonin in tumor cells [54–56]. In particular, Pariente et al [54–56] demonstrated that melatonin increased the production of ROS in cervical cancer HeLa cells. These results, taken together with our own findings suggest that melatonin may have a pro-oxidant role related to its concentration and the cell type.

Apoptosis, triggered by multiple cellular pathways, is a cellular self-destruction mechanism involved in a variety of biological events. Mitochondria play a key role in two of the better characterized pathways involved in apoptosis, the extrinsic and intrinsic pathways. Recent studies support the idea that mitochondrial fission has important physiological functions in cellular physiology such as the segregation of mitochondria into daughter cells during cell division and the distribution of mitochondria along microtubule tracks to the terminal synapses, where there is a high-energy demand [57, 58]. However, excessive mitochondrial fragmentation appears to play an important role in apoptotic death, including in neuronal cells [48, 59, 60]. Moreover, recent papers have shown how many of the biological effects of melatonin stem from its ability to selectively accumulate in mitochondria. For this reason mitochondria are considered the biological targets of melatonin [61, 62]. SD significantly increases DCFDA/MTRC positivity by four fold compared to controls and induces a marker release of cyt-*c* from mitochondria. ROS production leads to mitochondrial damage and apoptosis which occurs *via* the extrinsic or intrinsic pathway.

While the links between mitochondrial damage and apoptosis are well documented much less is known about the role of the mitochondrial network in relation to apoptosis, although Ong et al. [63] demonstrated that in ischemic reperfusion a pharmacological inhibitor of DRP-1 decreased cell death. In our study, apoptotic features are coupled with the fragmentation of mitochondria investigated by DRP1 and pDRP1. It is noteworthy that SD was found to induce DRP1 translocation from the cytoplasm to the mitochondria and that the FF values obtained from confocal analysis confirm a change in the length and degree of branching in the mitochondria. DRP1 was shown to be associated with proapoptotic members of Bcl-2 family proteins at the mitochondrial scission sites during the apoptotic process; however, neither the DRP-1 protein *per se* nor mitochondrial scission by itself has been shown to induce cell death. Conversely, several lines of evidence suggest that DRP-1 dependent mitochondrial fragmentation is an early event that leads to increased ROS production and final neuron death. Despite these discrepancies, it is clear that there is a close interaction among ROS, apoptosis and mitochondria fragmentation and our data are in line with the above-mentioned observations. This very much appears to be a chicken or egg dilemma as it is unclear whether ROS trigger mitochondrial fragmentation or *vice versa*. In our experimental conditions, we were not able to provide a chronological breakdown of different events because all the observations were made after 24h of treatment. However, we found that melatonin pre-loading restored the translocation of DRP-1 and preserved mitochondrial morphology, affecting the apoptotic pathway. As previously explained, SD treatment induces a cross-talk between apoptosis and autophagy. Autophagy is the catabolic process of cellular components, including cytosolic protein, aggregates and organelles such as mitochondria, which are sequestered in the autophagosomes [64]. The process of mitochondrial degradation through macroautophagy is called mitophagy. The role of mitophagy is the clearance of damaged mitochondria and acting in concert with mitochondrial biogenesis, it ensures a healthy mitochondrial network through mitochondrial turnover. The present study found that apoptosis was accompanied by a marked alteration in autophagy as shown by the LC3B-GFP analysis, as well as in mitophagy, a phenomenon involved in the quality control of mitochondria [65]. Elevated mitophagy has been observed in several neurodegenerative diseases whereas inhibitors of autophagy sometimes protect against axonal/dendritic degeneration or cell death associated with elevated mitophagy [65]. However, whether or not mitophagy depends on prior mitochondrial fragmentation promoted by DRP1 is controversial. In fact, Yamashita et al. [66] demonstrated that mitochondrial fragments start to bud and divide from mitochondrial tubules when in tight association with forming autophagosomes, but independently of the mitochondrial division factor Drp1/Dnm1. On the contrary, Kageyama et al. [60] showed that in the absence of mitochondrial division mediated by DRP1, mitochondria showed increased connectivity and size and became defective in mitophagy. In our experimental conditions, oxidative stress transformed mitochondrial morphology from elongated tubules to large spheres, as shown in Fig 3. It is important to underline that the spherical morphology destroys the proper mitochondrial surface area to volume ratio and changes the intramitochondrial distribution of matrix proteins and mitochondrial DNA. Such alterations probably compromise the electron transport chain complexes and respiratory function as our results showed. In this scenario, the reduction of oxidative stress (DCFDA/MTR positivity) by melatonin could contribute to maintaining mitochondrial homeostasis. Furthermore, it should be emphasized that the clearance of dysfunctional mitochondria is a critical step to limit cellular damage via ROS production and subsequent apoptosis. Zhang [67] strongly supports the emerging hypothesis that mitochondrial division maintains mitochondrial functions by promoting mitophagy in order to facilitate the removal of oxidative damage. Furthermore, Shirihai’s group [68] connects mitophagy with mitochondrial fission. In their paper, the authors observe that asymmetric fission of modestly damaged (i.e. partially depolarized) mitochondria produces one healthy (hyperpolarized) daughter to join the functional pool, and one depolarized daughter specifically targeted for mitophagic elimination [69]. Despite some discrepancies, our data support the hypothesis that autophagy plays a protective role by removing cellular components damaged during apoptosis. Moreover, we speculate that melatonin affects the autophagic pathway because it is able to act on the apoptotic pathway improving cell viability.

Finally, our study provides evidence pointing to the involvement of plVDACs in the autophagic/apoptotic process induced by SD in HT22 cell line, which is blocked by melatonin pre-loading. In normal cells, this type of channel has been found to be silent, playing a role in the maintenance of redox homeostasis, being a NADH-ferricyanide reductase. However, apoptotic stimuli have been shown to activate VDACs in the plasma membrane of neuronal cell lines, promoting anion efflux and affecting redox homeostasis. It is noteworthy that the activation of plVDACs has been hypothesized to be concurrent with mitochondrial cyt-*c* release and the opening of K^+^ channels [30]. Altogether these events would be able to induce a further activation of the apoptotic process. Likewise, VDAC activity blocking has been found to prevent apoptosis. Thus, based on these considerations and the results of the present study, we could speculate that the SD -induced disruption of the mitochondrial dynamics of HT22 cells may result in VDAC activation in plasma membrane and that melatonin pre-treatment, which restores the mitochondrial network, could prevent the plVDAC from contributing to the apoptotic/autophagic process.

## Conclusions

**Fig 8:**
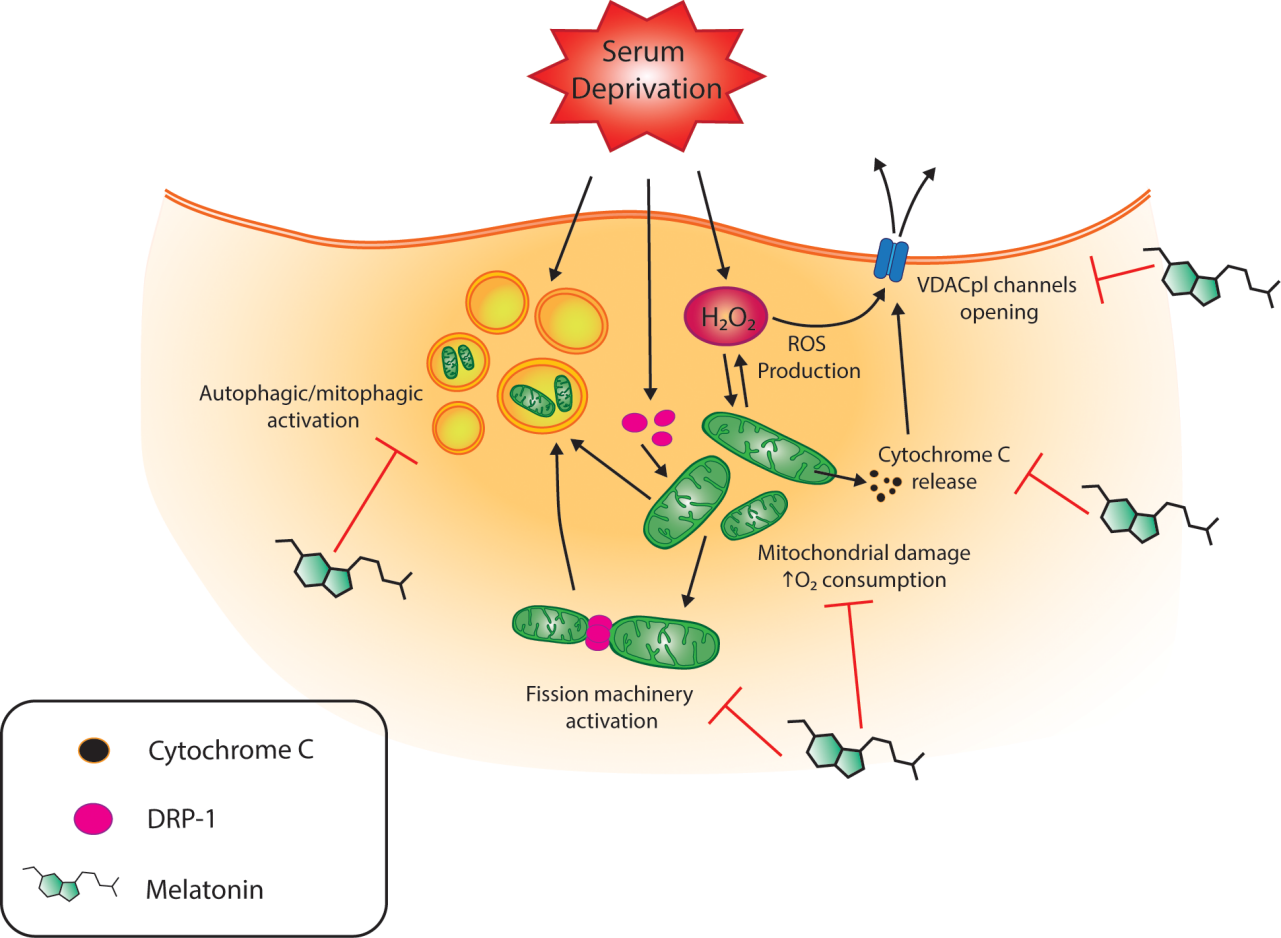
Schematic representation of the protective effects of melatonin against the action of serum-deprived HT22 cells. Serum deprivation leads to activation of the autophagic/apoptotic pathway, activation of the mitochondrial fission machinery and an increase in ROS. This in turn results in an increase in mitochondrial O_2_ consumption, cytochrome *c* release and the opening of plVDAC channels. Melatonin restores mitochondrial function acting on these pathways. Other details regarding the proposed effects of melatonin are reported in detail in the text.

The present study provides new insight into the neuroprotective role of melatonin, in particular, its effect on the apoptotic/autophagic pathway in HT22 serum- deprived cells. We show that melatonin has a wide range of positive converging effects on mitochondria under oxidative stress conditions. These findings, together with other cellular actions reported by several research groups pave the way for the administration of melatonin in the treatment of pathologies associated with an impairment of mitochondrial status (Fig 8).

## Supporting information

S1 Fig Expression of pDRP1. Cells were incubated with two different concentrations of MEL (200 and 500 μM) for 24 h before serum deprivation. The relative amount of pDRP1 proteins was quantified by densitometric analysis. Western blot analyses was carried out on three individual sample for each experimental condition; **P < 0.01 *vs* control, ^#^P < 0.05 and ^###^P < 0.001 *vs* SD. A representative blot is shown.
(TIF)
