# Supplementary figures and images for: Melatonin protects hippocampal HT22 cells from the effects of serum deprivation specifically targeting mitochondria
Source: PLoS One. 2018 Aug 29;13(8):e0203001. doi: 10.1371/journal.pone.0203001 (PMC6114848; doi:10.1371/journal.pone.0203001)

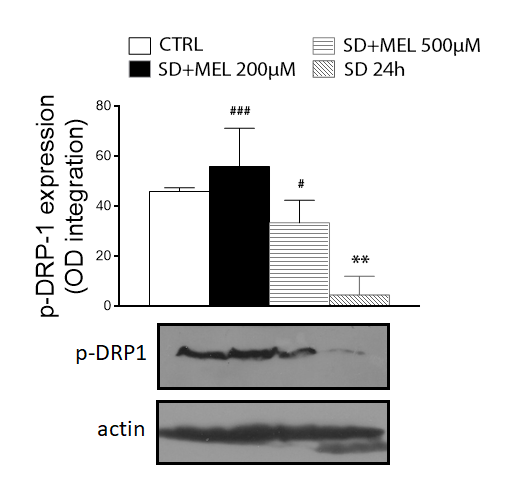

Supplement: S1 Fig — Cells were incubated with two different concentrations of MEL (200 and 500 μM) for 24 h before serum deprivation. The relative amount of pDRP1 proteins was quantified by densitometric analysis. Western blot analyses was carried out on three individual sample for each experimental condition; **P < 0.01 vs control, #P < 0.05 and ###P < 0.001 vs SD. A representative blot is shown. (TIF) [file pone.0203001.s001.tif]
